# An exploration of pregnant women and mothers’ attitudes, perceptions and experiences of formula feeding and formula marketing, and the factors that influence decision-making about infant feeding in South Africa

**DOI:** 10.1186/s12889-022-12784-y

**Published:** 2022-02-25

**Authors:** Christiane Horwood, Silondile Luthuli, Catherine Pereira-Kotze, Lyn Haskins, Gillian Kingston, Sithembile Dlamini-Nqeketo, Gilbert Tshitaudzi, Tanya Doherty

**Affiliations:** 1 Centre for Rural Health, University of KwaZulu-Natal, Durban, South Africa; 2 School of Public Health, University of the Western Cape, Cape Town, South Africa; 3 M&C Saatchi World Services, London, UK; 4 King’s Business School, Kings’ College London, London, UK; 5 World Health Organization, Pretoria, South Africa; 6 UNICEF, Pretoria, South Africa; 7 Health Systems Research Unit, South African Medical Research Council, Cape Town, South Africa

**Keywords:** Formula feeding, International code of marketing of breast-milk substitutes, Marketing, Infant feeding practices, Breast-milk substitutes, Child health, Global health, South Africa

## Abstract

**Background:**

Despite strong evidence showing the lifelong benefits of breastfeeding for mothers and children, global breastfeeding practices remain poor. The International Code of Marketing of Breastmilk Substitutes is an internationally agreed code of practice, adopted by the World Health Assembly in 1981, to regulate promotion of commercial formula, and is supported by legislation in many countries. However, marketing of formula remains widespread and contributes to mother’s decisions to formula feed. We present South African data from a multi-country, mixed-methods study exploring women’s decision-making about infant feeding and how this was influenced by exposure to formula marketing.

**Methods:**

Using a consumer-based marketing approach, focus group discussions (FGDs) were conducted with pregnant women and mothers of children aged between 0 and 18 months in two urban sites in South Africa. Participants were purposively selected according to their child’s age, infant feeding practices and socioeconomic status. Ten FGDs were conducted during February 2020 with a total of 69 participants. Thematic analysis was used to analyse the data with NVivo v.12 software.

**Results:**

Despite being encouraged by health professionals to breastfeed and intending to do so, many mothers chose to give formula in the early weeks and months of their child’s life. Mothers reported breastfeeding challenges as the most frequent reason for initiating infant formula, stating that family members and health professionals recommended formula to solve these challenges. Although participants described few advertisements for infant formula, advertisements for ‘growing-up’ formulas for older children were widespread and promoted brand recognition. Mothers experienced other marketing approaches including attractive packaging and shop displays of infant formula, and obtained information from social media and online mothers’ groups, which influenced their choice of formula brand. Mothers reported strong brand loyalty derived from previous experiences and recommendations. Health professionals frequently recommended formula, including recommending specific formula brands and specialist formulas.

**Conclusion:**

Global formula companies use multifaceted marketing methods to promote a strong narrative portraying formula feeding as a positive lifestyle choice. Positive, coordinated efforts are required to counter pro-formula messaging and change the narrative to support breastfeeding as an aspirational choice. In particular, health professionals must stop supporting the formula industry.

**Supplementary Information:**

The online version contains supplementary material available at 10.1186/s12889-022-12784-y.

## Introduction

There is overwhelming evidence of the wide-ranging, lifelong benefits of breastfeeding for both mothers and their children. Breastfed children have better health, nutrition and developmental outcomes in childhood compared to children in their communities who are formula fed [1–3]. Breastfed children grow into adults who are more successful, socioeconomically and academically [4, 5], with reduced risk of developing obesity and chronic diseases in later life [6]. As a result, infant and young child feeding guidelines unequivocally recommend breastfeeding as the best infant feeding method in all situations and settings worldwide [7]. Current WHO guidelines recommend exclusive breastfeeding for 6 months followed by the addition of nutritious complementary feeds and sustained breastfeeding up to 2 years of age and beyond [8].

However, in spite of these guidelines, rates of optimal breastfeeding remain low, resulting in a huge burden of preventable morbidity and mortality and loss of socioeconomic potential [9]. Although most women initiate breastfeeding, most do not breastfeed exclusively or sustain breastfeeding for the recommended duration. A review of national surveys in low- and middle-income countries (LMICs) showed that among infants younger than 6 months only 37% were exclusively breastfed, and among children aged 6-24 months, 37% were receiving no breastmilk at all [10]. These observations have led to major global initiatives to support breastfeeding, most recently the United Nations Decade of Action for Nutrition (2016-2025) [11].

In South Africa, optimal breastfeeding rates have been among the lowest in Africa [12]. In 2011 the Tshwane Declaration of Support for Breastfeeding was a national statement in support of breastfeeding, that sought to draw a line under previous policies that were not supportive of breastfeeding [13]. Support for breastfeeding has been backed by a series of government policies and guidelines including the Side-by-side initiative, MomConnect and Mother-baby friendly initiative [14–16]. Despite these efforts the most recent national survey, conducted in 2016, estimates that just 32% of infants aged below 6 months in South Africa are exclusively breastfed [17].

It has long been recognized that marketing of breast-milk substitutes has a pervasive effect on infant feeding decision-making and contributes to mothers’ decisions to choose sub-optimal feeding for their babies [9, 18]. Large powerful global corporations who manufacture commercial formula products use emotive appealing marketing strategies, targeted messaging, advertising and development of brand identity to encourage mothers to feed their babies with formula [18–21]. In order to reduce the influence of companies seeking to promote commercial formula, the International Code of Marketing of Breast-milk Substitutes (known as ‘The Code’), an international health policy framework for breastfeeding promotion, was adopted by the World Health Assembly (WHA) of the World Health Organization (WHO) in 1981 [22]. The Code seeks to prevent companies advertising or promoting breast-milk substitutes for babies aged below 36 months, to avoid conflicts of interest among health practitioners by preventing companies from providing incentives, thereby removing marketing pressures from discussions about infant feeding.

However, despite adoption of the Code in 136 countries [23], global sales of breast-milk substitutes have an annual value of over US$ 44 billion which is increasing year-on-year [9]. Marketing companies continue with innovative targeted marketing, taking advantage of the expansion of social media and online mothers’ groups [19, 20], and more recently, taking advantage of the COVID-19 epidemic to increase marketing activities in violation of the Code [21]. In South Africa, the Code was legislated through wide-ranging Regulations Relating to Foodstuffs for Infants and Young Children (R991) in 2012 [24]. The R991 regulations prohibit all types of commercial formula promotion including advertising of commercial formula products for children aged below 36 months, formula samples or gifts of any kind, promotional devices at points of sale, and any contact between formula companies and mothers. Any promotion of formula in health facilities is also prohibited [25]. However, despite measures for enforcement being in place, violations of the Code remain common in South Africa [26].

Low breastfeeding rates indicate that most women sooner or later choose to give their babies breast-milk substitutes, usually commercial formula. In this paper we explore women’s perspectives of the reasons for using formula, factors that influenced decision-making, and describe women’s perceptions and experiences of formula marketing and formula feeding in South Africa.

## Methods

We report findings from the South African component of a multi-country study conducted in eight countries using consumer-based marketing methodologies to explore women’s perceptions and experiences of decision-making about infant feeding, and the factors that influenced their feeding choices. In particular, we explored exposure to marketing messages about formula feeding and the influence of these messages on mothers, health professionals and other stakeholders. A consumer-based marketing strategy aims to explore the responses of consumers to marketing messaging using a variety of data collection methods, including product testing and marketing diaries [27]. The multi-country study comprised a series of Focus Group Discussions (FGDs) and in-depth interviews conducted with mothers, family members and health professionals; a survey among pregnant women and mothers of young children; and marketing diaries completed by participants. Participating countries were South Africa, United Kingdom, Vietnam, Nigeria, Bangladesh, Morocco, China and Mexico. The research was commissioned by WHO with the explicit intention of using marketing approaches to provide novel insights into infant feeding decision making. Research methods were overseen by an international panel of infant feeding research experts [28].

In this manuscript we report data from a series of FGDs conducted with mothers and pregnant women in two urban sites, Johannesburg and Cape Town, South Africa.

### Selection of participants

Women were selected based on their current feeding practices: breastfeeding only; formula only; or both breastmilk and formula. In addition, groups were stratified based on pregnancy or the age of the baby (either 0-5 months or 6-18 months) and socioeconomic status (either high or low). High socioeconomic status (SES) was defined as household income of > R15000 (approx. USD 1000) per month, and low SES was defined as < R15000 per month.

Convenience sampling techniques were used. Trained recruiters were deployed to health facilities in low, middle and high-income areas in Johannesburg and Cape Town. Women were approached either in the health facility or in the surrounding area including in the street, shopping malls, pharmacies, etc. Recruiters wore badges from the marketing company, they introduced themselves and provided scripted information about the study. In addition, participants were requested to identify other eligible participants from among their social network, this sampling technique is known as snowball sampling.

When a woman expressed interest in participating a screening tool was used to determine eligibility and record key characteristics (age of baby, race, SES, etc). Eligibility assessment included questions to ensure that the potential participant’s literacy level was sufficient to read written messages on packaging and advertising. Contact details of eligible participants were collected, and used by researchers to assign participants to the groups according to the specified characteristics. Participants were then contacted and requested to participate.

### Data collection

All FGDs were overseen by M&C Saatchi World Services and conducted by a local partner agency in South Africa (KLA), who specialises in market research. FGD moderators were all female and were trained by a member of the M&C Saatchi research team. These training sessions provided background to the study, covered recruitment and screening processes and provided an overview of ethical considerations for the study. FGDs were conducted in person and were moderated by one moderator who was the same race as participants. An observer was present to oversee the process.

Discussion guides, developed by M&C Saatchi World Services, were pretested, piloted and adapted as necessary to ensure that open questions and appropriate probes were used (supplementary file 1). Topics covered were sources of information about infant feeding; what influenced infant feeding decisions; attitudes and experiences of formula feeding; review of products and packaging. FGDs were designed to be interactive, and product testing was undertaken to uncover participants’ thoughts and feelings toward brands, products, and marketing. Product testing involved showing formula tins from different brands available in South Africa for participants to look at and discuss their knowledge and views about different brands. These techniques allowed the moderator to explore emotions and aspirations relevant to formula and infant feeding practices, central themes appearing in conversations around formula feeding, and how marketing messages related to women’s perceptions of formula. FGDs were conducted in English and were conducted in local office spaces.

### Analysis

All FGDs were audio recorded and transcribed verbatim. A thematic approach was taken to analyse the transcript data. Thematic analysis allows a flexible approach for reporting the perspectives of different research participants and summarising key features of a large dataset [29]. Two researchers (SL, CH) independently read and re-read the transcripts and developed a priori themes based on the research questions, as well as new themes that emerged from the data. These were discussed between the two researchers and the wider research team until any discrepancies were resolved and themes were agreed upon. Analysis was undertaken using NVivo v12.

## Results

Ten FGDs were conducted with women in Johannesburg (5) and in Cape Town (5) during February 2020. Participants were placed in groups according to race, socioeconomic status, age of the baby and feeding practice as shown in Table 1. We report on the following key themes: how women made decisions about feeding their baby; reasons for choosing to give formula feeds; influencers who were important in the decision to give infant formula; the process of choosing a particular brand of formula; and experiences of starting infant formula.

**Table 1:** Characteristics of Focus Group participants

#	Site	Group	Socio-demographics	IYCF practices	# participants
C1	Cape Town	Mothers of infants 0-6 months	Black; low SES	Exclusive or predominant breastfeeding	7
C2	Cape Town	Pregnant women	All races; high SES	First pregnancy (2) Older children (5)	7
C3	Cape Town	Mothers of infants 6-18 months	All races; high SES	Breastfeeding then introduced formula	7
C4	Cape Town	Mothers of infants 0-6 months	Black; low SES	Breastfeeding then introduced formula	7
C5	Cape Town	Mother of infants 0-6 months	All races; high SES	Formula feeding from birth	6
J1	Johannesburg	Pregnant women	Black; low SES	First pregnancy (3) Older children (4)	7
J2	Johannesburg	Mothers of infants 0-6 months	All races; high SES	Exclusive or predominant breastfeeding	6
J3	Johannesburg	Mother of infants 6-18 months	Black; low SES	Initiated breastfeeding then added formula	8
J4	Johannesburg	Mothers of infants 0-6 months	All races; high SES	Initiated breastfeeding then added formula	7
J6	Johannesburg	Mothers of infants 0-6 months	Black; low SES	Formula feeding from birth	7

### Decision making about infant feeding

Most women planned to initiate breastfeeding, even if it was for a short period, and very few women reported that they had intended to formula feed from birth. Even in groups where mothers were selected based on formula feeding from birth, many of the mothers had planned to breastfeed but were unable to do so. Women in all groups reported that they were encouraged to breastfeed by health professionals in both the private and public sector, particularly during pregnancy. Women indicated that the strong message was always ‘*breast is best’* and that the benefits of breastfeeding were the primary reason for planning to breastfeed.

*At the clinic it’s almost like they discourage formula feeding because every time you go there for your antenatals at the clinic you will get a 5-minute session where they will be talking about the importance of breastfeeding and urging us to breastfeed (FGD C4, mixed feeding)*

However, women mentioned several perceived advantages of formula feeding that they considered when making their feeding choice, including perceptions that a formula fed baby was more settled, slept better, gained more weight, and that the baby’s father could help with feeding. However, the high cost of formula was an important factor that women considered, which discouraged some women from formula feeding.

*My friend’s baby was being fed S26 Gold and I looked at the price and I was like, this is mighty expensive and there is just no way that I am going to spend my money on that, so I decided to breastfeed instead (FGD C1, predominant breastfeeding)*

Women usually perceived the method of feeding their baby as being a choice between two comparable options, and some women strongly emphasized that the feeding method should be their choice. Women complained that they were given little or no information about infant formula, and requested more information be provided, suggesting that mothers should be given more of a balanced choice.

*I don't know if it’s what you want to hear but I’d like the pros and cons of both (breast and formula) to be advertised just as much, so that when you’re making your decision there’s no preference, one to the other. One is always going to have different advantages to the other. So, if there can be a range of comparisons between one and the other, so you can identify what those advantages and disadvantages are and make a clear decision so that you’re not pushed to one – this is the best. (FGD C2, pregnant)*

A few women expressed anger on being informed that advertising is strictly regulated and formula companies are not allowed to advertise, saying that this made getting information difficult. A number of women mentioned that they felt pressured to breastfeed, and that women who formula fed were looked down on and made to feel like bad mothers.

*At the hospital they asked if I was going to be breastfeeding or giving the baby formula? And you know how our black sisters will be like (making shocked noises), “She is going to be bottle feeding the baby” … They don’t want to give you formula in hospital, they really don’t (FGD J4, mixed feeding)*

Further, a number of women expressed the view that relatives and friends had been formula fed and were fit and healthy, suggesting that the benefits of breastfeeding were overstated, particularly mentioning that breastmilk could be adversely affected by the mother’s diet and lifestyle. Several women stated that there was little difference between the nutrition from breastmilk and formula, and a few women expressed that because formula was ‘scientifically formulated’ it might actually be healthier than breastmilk.

*I get breastfeeding is best for your baby but the nutrition you get in your breastmilk is the exact same thing, and sometimes you get more vitamins out of actual formula feed than what you get out of breast milk because your breast milk is also dependant on how you’re eating, your diet and your (indiscernible). So if you’re not sleeping, you’re tired and drained, your baby’s not getting all the nourishment. (FGD C2, pregnant)*

### Why do mothers initiate formula feeds?

Despite initially planning to breastfeed, many women made the decision to add formula or stop breastfeeding in the days, weeks and months after delivery, most often because they experienced challenges with breastfeeding. In the first few days of the baby’s life many women struggled to establish breastfeeding. A number of women vividly described their bad experiences and the stress of being unable to latch with a crying baby. ‘*I won’t lie, with my first baby, it was probably the most traumatizing thing, it wasn’t the giving birth or anything, it was just the breastfeeding’ (FGD J4, mixed feeding).* It was common for health facilities in both private and public sectors to routinely ‘top-up’ with formula in this early period.

*In hospital they do recommend breastfeeding, but because … he wasn’t latching properly, we went through the process where the nurse came in, assisted me with latching and stuff. When that didn’t work, they would then give him a bit of formula, so we did a bit of both in hospital (FGD C3, mixed feeding)*

Once breastfeeding was initiated, breastfeeding challenges continued to be the main reason for starting formula, including pain while breastfeeding, perceived insufficient milk and inadequate weight gain. Very few mothers reported being given support and advice to address these challenges while continuing to breastfeed. Instead, adding formula was seen as the solution by the mothers themselves, their families and by health professionals.

*I also tried breastfeeding at the beginning. I breastfed for about a month, it was very difficult, it was incredibly painful, he just wasn’t satisfied, and when I went to the pediatrician, she explained that maybe it would be a better idea to try formula, which we did (FGD J4, mixed feeding)*

It was common for women with breastfeeding challenges to report relief that when they added formula the struggles with breastfeeding were resolved. Several women described advantages of starting formula feeding as follows:

- *R1: You worry less.*
- *R2: They sleep longer.*
- *R3: My favourite one is my husband takes the night feed. I love it. (FGD C5, formula feeding)*

Another common reason for early cessation of breastfeeding was returning to work, with many working women saying they were not supported to express and store breastmilk in the workplace ‘*My boss told me straight up, “Please don’t think you are going to get time off to do your lady boob things”, and I was like, thanks’ (FGD J4, mixed feeding)*. Another factor mentioned by mothers was that breastfeeding limited their activities *“you can’t do anything because (of) this child, you are (like) a walking … fridge” (FGD J1, pregnant).* Women did not want to be tied to the baby all the time, perceiving formula feeding as more convenient.

### Who influences the decision to give formula?

Women often turned to others for advice about feeding. Family members were important in influencing women to start formula feeding, with many women reporting being put under pressure by family members, particularly their mothers and grandmothers, to add formula if the baby appeared hungry or was crying or feeding frequently.

*Your family (advises you) because sometimes you have to run some errands and leave your baby behind or sometimes the child will just cry all night long even though you are giving her breast milk, you understand? So, they will say you must add on formula so that the child can sleep through the night (FGD C4, mixed feeding)*

Health practitioners were an important influence on decision making, mothers mentioned lactation specialists, clinic nurses, pharmacy workers and doctors, mainly in the private sector, had advised them to formula feed. In particular, doctors and paediatricians were strongly valued as experts, and mothers often reported that advice from the doctor was the primary reason for starting formula feeding.

*I think I just got the best news on the planet, when she (pharmacy nurse) said, “Stop doing this to yourself mommy, put your baby on formula, come let me take you”, and she literally stood there with me in front of that aisle … Every single formula she pointed to, right, this one is good for the tummy, this one is good for (trails off). I was like wow. And she was like, I recommend NAN, she gave me a little tin, and we went, and she even phoned me a week later, how you doing? (FGD J4, mixed feeding)*

### How do mothers choose a formula brand?

Having decided to give their baby formula, women described being faced with deciding which brand to use. This was often based on brand recognition, previous experience or recommendations. Some women mentioned that they were influenced by the appearance of the tins of formula in the shop and how prominently the tins were displayed. Women highlighted things that attracted them to a particular brand, some mentioned more appealing packaging, others that the names suggested that some brands were better than others, for example *gold* in the name or the word *premium* suggested that these were better brands.

*It’s so pretty (the packaging), and I know it sounds bad, but my mommy instinct took over and I wanted the most expensive, because I am making up for not breast feeding her (FGD J4, mixed feeding)*

Most women agreed that there were very few overt advertisements, for example in magazines, on the radio or on television, or special prices for infant formula. However, some women were attracted by advertising for formula for older children (known as growing-up milk). Although not aimed at infants, mothers received information from these adverts in relation to brand recognition, and advertising messages about the benefits of formula, as described by this mother:

*When I was in the store looking at so many, I recognised Nan and I don't know where from, I feel like it’s got way more of an online presence, especially on TV and that’s why it’s stuck in my memory and I don't recognise the other ones. That’s why I felt safe going with Nan. (FGD C3, mixed feeding)*

Although women did not report seeing formal advertisements for infant formula, all women were exposed to various other types of marketing. Many mothers described accessing information from online sources including Instagram and Facebook as well as from online mother’s groups. All women, including those who were breastfeeding, discussed at length their perceptions about the various brands of formula. Many women expressed strong opinions that some commercial formula products were ‘better’ than others. It was common for women to state that the best formulas were those described as being most like breastmilk.

*Actually, for me it was a doctor who told me that I should use Pelargon as it is light and therefore similar to breast milk. The doctor was actually saying even if the mom is not breastfeeding the child, if they are giving them Pelargon it’s actually tantamount to giving them breast milk (FGD J3, mixed feeding)*

Women also discussed in detail differences in prices and composition of formula brands. Although affordability was a factor in choosing, most women from both high and low socioeconomic groups felt that more expensive formula brands were better, and they did not want to choose those formula brands that were considered to be ‘cheap’.

*I just like the idea of it being natural, the one thing I do is read the ingredients on these formulas. And when I looked at the products from overseas, they ask what is the closest part to breastfeeding. We don’t have a lot of formulas that are closest to breastfeeding (FGD J4, mixed feeding).*

However, there was also a perception that expensive formula brands were more ‘rich’ than others, and might cause problems, ‘*they ended up having some chest problems like short-breath and all that because of having too much rich milk*’ *(FGD C1, breastfeeding),* so that despite being more expensive, these brands may not be the best for your baby. Many women described brand loyalty, having used a particular brand before or suggesting that a particular formula brand was always used in their family.

*I heard that it’s a generation thing, my cousin who is 30 years old now was drinking that (formula) milk, apparently I was drinking that myself and when I was pregnant my mom also told me that when I start giving my baby formula it is what I must use. It’s quite expensive though from what I have seen in the shops (FGD C1, breastfeeding)*

Similarly, recommendations from friends or family were important in choosing the formula brand. Many women described that the experiences of others using a particular formula brand was what motivated them to choose that brand for their baby.

*My cousin has triplets. And I’ll see her kids are growing well … that’s actually one of the reasons I chose the formula that my eldest son was on, because her son was on it as well and he grew well, he reached his milestones (FGD C3, mixed feeding)*

Numerous women reported being advised by health professionals, particularly doctors and paediatricians, about which brand to choose or which brand was most suitable for their baby. There was a strong narrative around so-called ‘specialist formulas’ recommended by health professionals for problems like allergies, colic or reflux, and mothers reported being advised to add formula or change brands to manage babies with these conditions.

*Well, my paediatrician only spoke Nestle products so I’ve only known Nan and Lactogen, so with my son now I did the same start process, he was on the allergy and then on Nan for two months and then Lactogen. I switched to Lactogen because it’s cheaper, it’s like R100 cheaper than what Nan is and it’s the same product, same company (FGD C3, mixed feeding)*

*So, the doctor told me if I try formula milk I would rather go for S26 because they were also checking my baby’s health. Like for now he has a lot of diarrhoea – I don’t know why … I don’t know if it is all the hospitals that use S26 (FGD J2, predominant breastfeeding)*

Another deciding factor that women mentioned was the brand that was given to them at the hospital, in several cases this was the brand they chose when discharged.

*It also comes highly recommended because when you’ve just delivered your baby the hospital uses Nan. And if the lactation process is happening very slowly for you they will give you some Nan in a small cup to tide you over in the meantime. So, I think that’s why most people just go straight to Nan (FGD J3, mixed feeding)*

### Mothers’ experiences of starting formula

Many women spoke of their experiences starting formula feeding, describing both positive and negative experiences. Although for mothers experiencing breastfeeding challenges starting formula was often experienced as the solution to their problems, there was also a strong narrative about negative effects of starting formula, with many women describing that formula made their children ill, giving them diarrhoea, colic or rashes. In most cases this was not perceived as being the result of starting formula but rather that the particular brand chosen was not the ‘correct’ one for the child. Many women described a process of trying many different formula brands before finding the one that was perceived as most suitable for the child, including visiting a series of health professionals to get advice on which brand of formula to choose.

*So, I took her to the paediatrician in Milnerton. She said it was reflux and … . I took her to the next doctor and I am going back and forth, back and forth. Then it was just the reflux. I had her on Lactogen, all the milks you can think of. The Pelargon make her tummy very hard and she was crying and just build up. The Simalac did the same. (FGD C5, formula feeding)*

## Discussion

Our study shows that despite most women planning to breastfeed, it is common for women to start formula, and for health professionals and family members to recommend formula feeding as a solution to perceived feeding difficulties. This is despite the problems associated with initiating formula described by many women and the overwhelming evidence of the crucial benefits of breastfeeding. The narratives used by women to explain this decision are strongly resonant of the messages promoted by the global formula companies: that formula is similar to breastmilk; that formula feeding is a lifestyle choice; and that mothers have the right to choose what they perceive is best for their baby according to their circumstances. Similarly, health professionals provided messages that mirror messages from formula companies, reinforcing a strong narrative that there are important differences between formula brands and recommending different formula brands for problems associated with formula feeding, for example diarrhea, colic and allergies.

Although there were few examples of overt advertising of formula for infants or for children aged under 36 months, women were exposed to other marketing strategies including packaging and presentation of formula in shops. Mothers were influenced by the composition of different formula brands written on the tin, particularly when they perceived that the formula contained ingredients that were “similar to breastmilk”, and were influenced by product names, with certain words being interpreted as suggesting that some formulas were better. In addition, the advertising of brands of ‘growing-up milk’ for older children was used to promote brand recognition that influenced choices of infant formula brand for younger children and infants. This suggests that current limitations to advertising of formula in the South African R991 legislation are insufficient to prevent other marketing strategies, and marketing messages continue to play a strong role in infant feeding decision-making. We suggest that neutral packaging for formula for infants and children, as well as regulating and standardizing how tins of formula are presented in shops would strengthen implementation of the Code in South Africa and elsewhere. In addition, formula used in both public and private hospitals should be presented in non-branded packaging. Many women did not support the legislation to limit advertising of formula, even suggesting that this puts them under undue pressure to breastfeed, so we suggest that stronger regulations be accompanied by an innovative market orientated communication strategy to improve understanding about the negative effects of formula on child health, nutrition and development.

Furthermore, it is clear that the current approach of simply discouraging use of formula and encouraging breastfeeding has failed to convince most mothers. In the current environment of social media, it is impossible to fully control the marketing from formula companies. Formula manufacturers have access to huge resources and high-quality marketing skills, and can use a variety of insidious and covert marketing methods to get their messages across to mothers and health workers [20]. The views of the women in our study suggest that these messages are received by mothers and communities, and that they influence feeding practices. It is possible that the failure of health services to directly challenge these messages may allow the formula companies to dominate the narrative, particularly in women’s groups and on social media. Specific communication strategies should be developed to discredit pro-formula messages and counter the narrative that formula feeding is convenient and aspirational for modern women, where breastfeeding is old fashioned and messy. Communications strategies should learn from the strategies of marketing companies and use innovative messaging to portray breastfeeding as aspirational and suitable for mothers who want their children to be smart, healthy and to be high achievers in life. All mothers, families and communities want the best for their children, and given the substantial benefits of breastfeeding it should be possible to counter the insidious pro-formula messaging propagated by global marketing strategies. Further research is required to evaluate strategies to inform women about formula, change their attitudes and practices, and support positive breastfeeding messages. There are a number of existing strategies supporting breastfeeding in South Africa, which could be strengthened to directly counter pro-formula messages [14–16].

Another key finding from our study was the crucial role played by health professionals in medicalizing normal infant behaviours, and spreading messages that breastfeeding problems are best solved by formula feeding, and problems with formula feeding are best solved by a different brand of formula. The majority of such problems are best solved by breastfeeding, and health professionals have an ethical responsibility to provide support for women to continue breastfeeding. Challenges with breastfeeding were often fundamental to mothers deciding to initiate formula feeds, and mothers most often turned to health professionals for advice in this situation. Health professionals are highly respected as experts, so their views are particularly influential. Many health professionals engage with representatives of companies marketing commercial formula products [30], and our findings suggest that marketing strategies successfully influence health professionals to provide messages supportive of formula feeding. These practices are directly contrary to all relevant national policy regulations, including the Tshwane declaration, South African infant and young child feeding policy, the R991 regulations, as well as being in violation of the Code. Further training and support could encourage health professionals to align their practices with national policies, provide information about ethical practice, and practical strategies to support mothers to address breastfeeding challenges. Professional bodies have a role to play in encouraging health professionals to act ethically and according to national and international policies supporting breastfeeding, and in particular, discouraging practitioners from engaging with commercial formula companies and their representatives.

This study employed a strong qualitative design and included a diverse range of socioeconomic backgrounds with participating women who attended both private and public health facilities, giving a wide range of perspectives. However, only urban women were included and lower socioeconomic groups were relatively affluent by South African standards. FGDs were conducted in English and anyone who was illiterate in English was excluded. Thus, the views of women with very low income, rural women and non-English speaking women were not heard.

## Conclusion

This study goes some way to explain how and why women in South Africa make the choice to feed their children with a product that leads to ill health and reduced cognitive development. Strong, coordinated efforts are required to actively counter the arguments from formula companies that portray formula feeding as a positive lifestyle choice. Further, health practitioners need to recognize their pivotal role in promoting formula feeding as opinion leaders in the field, and provide mothers with support to continue breastfeeding.

## Supplementary Information

**Additional file 1.**

## Data Availability

All data, transcripts and study tools to support the findings of this study are available from the Centre for Rural Health and will be made available upon reasonable request from the principal investigator or corresponding author. The data is not available publicly because analysis is ongoing and further manuscripts are being written.

## Abbreviations

ERC: Ethics Review Committee
FGD: Focus Group Discussion
SES: Socioeconomic Status
USD: United States Dollar
WHO: World Health Organization
