# Supplementary material for: An exploration of pregnant women and mothers’ attitudes, perceptions and experiences of formula feeding and formula marketing, and the factors that influence decision-making about infant feeding in South Africa
Source: BMC Public Health. 2022 Feb 25;22:393. doi: 10.1186/s12889-022-12784-y (PMC8872897; doi:10.1186/s12889-022-12784-y)
Supplement: Supplementary file 1 — Additional file 1. [file 12889_2022_12784_MOESM1_ESM.pdf]

M&C SAATCHI
WORLD SERVICES
RESEARCH, INSIGHT & EVALUATION

# DISCUSSION GUIDE

# FOCUS GROUP DISCUSSION WITH WOMEN

**FEBRUARY 2020**

**FOCUS GROUP DISCUSSIONS WITH WOMEN**

**INSTRUCTIONS FOR MODERATOR**

It is important that all the areas pertinent to the objectives are covered in the focus groups, you may use additional probes if suitable. If the discussion moves away from the topic outlined, guide the conversation back to the main questions. It is important to stick as closely as possible to the guide times given to cover each area of the discussion.

Each group participant should have the opportunity to speak. Encourage all participants to respond to each main question, even if just to confirm that they have nothing further to add.

Participants will have been provided with the participant information sheet to read before agreeing to take part. As participants enter the room, you should give them a second copy to re-read. Please provide support with this and respond to any questions the participants might have.

You should audio record the focus group using at least two recording devices. For the group tasks place an additional recording device on each table.

**KEY OBJECTIVES**

- Understand the main sources of information and the influences on infant feeding – what and who?
- Understand what people think about breastfeeding and formula feeding- benefits and barriers/ strengths and weaknesses
- Understand why people choose formulas, what are the beliefs around claims of formula? What makes formulas more or less appealing?
- Understand whether women think there is a need for stage 2/3/ and 4 formula (follow on/ toddler milks) and why?
- Understand women's views on advertising and exposure to advertising- especially below the line advertising

## SECTION ONE: INTRODUCTION AND WARM UP [15 MINUTES]

**MODERATOR - READ THE FOLLOWING**

*Thank you for attending this discussion today. Before we begin, I would like to set out a few ground rules. We want to make sure that everyone feels comfortable to share their thoughts and experiences, so I would like to draw your attention to the following:*

*Everyone gets a chance to participate and share their thoughts and experiences. Some of us naturally have more to say than others but I want to make sure today that everyone gets a chance to speak and feels confident enough to do so. So, I will sometimes encourage specific individuals to share their thoughts and experiences.*

*Everyone respects each other's thoughts and experiences. There are no right or wrong answers, and everyone has different experiences in their lives that lead them to make the choices and decisions that they do.*

***Everything we say during this discussion should remain in the room. I ask you to respect each other's views by not sharing them outside of this room. I would like to remind you that you have agreed to this in your consent form.***

*Is everyone happy with these rules?*

**MODERATOR - CHECK THAT EVERYONE IS IN AGREEMENT**

*Thank you.*

## INTRODUCTION – 5 MINUTES

*The focus of our discussion today will be pregnancy and motherhood. Now let's start by getting to know each another a little better. Please tell the group a little about yourself and your baby/ pregnancy. For example*

- What is your name and where do you come from?
- What is your baby's name and how old are they/when is your baby due?
- Do you have any other children?
- Can you tell me about how you feed your baby/ plan to feed your baby?
- How or why did you decide to feed/ plan to feed your baby this way?

## SECTION TWO: INFORMATION SOURCES- 15 MINS

**MODERATOR**

*Now I'd like to discuss as a group the information sources that you use relating to motherhood, feeding your baby and parenting.*

1. What are the sources that you use for information about breastfeeding and formula feeding?

2. Who are/were the most influential people when making the decision as to how to feed your baby?

3. Did anyone recommend that you breastfeed, or formula feed your baby? Family, friends, other mums etc

4. What about health professionals or other 'experts'? Do you go to any for information on how to feed your baby? Have they been influential in your plans/ current feeding regime?

**We now want to talk about the online resources that you use to get advice and information about infant feeding, talk to other mothers e.g. Facebook, Instagram, WhatsApp etc.**

5. Do you use online resources for information on infant feeding? What about aps, mum's forums, influencers, bloggers, etc? If so how do you use these, how often?

6. What do you like, dislike about these? Do you find them useful?

## SECTION THREE: ATTITUDES TO INFANT FEEDING- 15 MINS

**NOTE TO MODERATOR IF FEEDING BEHAVIOURS OF POSITIVE HIV IS MENTIONED PLEASE PROMPT**

7. At what point do you think that most women decide how they want to feed their baby? Is it when they are pregnant? once the baby is born?

8. Who or what do you think influences their plans and decisions on how most?

9. What do you think is the best way to feed a baby aged 0-6 months?

10. And what about an older baby aged up to 12 months, what should you feed them?

11. If a woman is HIV positive, what do you think is the best way for her to feed her baby?

12. Now thinking about different types of milk (including breast milk, cow or goat milk, formula milks etc.). What types of milk are best to feed an infant at 0-6 months? 7-12 months, 12-18 months, more than 18 months?

13. If a woman uses formula how long do you think she should use it for?

14. How do you think most women feed their babies – breastfeed, formula, follow on milk etc? And do you think this has changed since your parent's generation?

## SECTION FOUR: BREASTFEEDING- 10 MINS

15. What do you think about breastfeeding? What are your thoughts and experiences? How easy did you find breastfeeding?

16. Do you think that most women can breastfeed if they want to?

17. What do you think are the main reasons why women choose to breastfeed?

18. What do you think are the main barriers that prevent or stop women from breastfeeding?

19. Do you think that the diet that women eat influences the quality of their breast milk?

## SECTION FIVE: FORMULA FEEDING- 10 MINS

20. What do you think about formula feeding? What are your thoughts and/ or experiences?

21. What do you think are the main reasons why women choose to formula feed?

22. Why do you think women introduce formula from birth, or switch to or introduce mixed feeding?

23. And what do you think about mixed feeding?

## SECTION SIX: PRODUCTS SECTION- 20 MINS

**FIRST UNPROMPTED WITHOUT SHOWING PRODUCTS**

24. What brands of formula are you aware of? Which ones would you/do you use?

25. If you do use formula, why did you choose to use the brand of formula you use? Who or what influenced your decision on brand?

26. Has anyone ever recommended a brand of formula to you? For example a health professional or friend?
    - Prompt if health professional- where? When? What did they say?

27. Where do you get information on what type of brand of formula to use?

**THEN SHOW PRODUCTS**

28. Which have you seen before, which have you used?

29. is it clear what each are for, in terms of age, special conditions?

30. Have you seen any of these? Have you used them? Prompt if not mentioned – specific formulas for allergies, organic, closer to breastmilk etc,

31. What do you think of the developments in formula?

32. Do you think any of these products are superior? If so why?

33. Where do you learn about formula and which types to use?

34. Which brand do you think is best?
    -why is this?

35. What do you think about the price of formula?

**NOTE TO MODERATOR TO PROBE IF ANY MENTION OF PRODUCTS BEING SIMILAR TO BREASTMILK**

## SECTION SEVEN: FORMULA BRANDS AND MARKETING- 15 MINS

36. Have you seen any advertising for formula brands recently? TV, posters, social media, other online ads? Where? What did they ad say? For which brands?

37. What do you remember about these ads, what were they saying about the brands, products?

38. Have you seen any promotions for formula, instore or online?

39. Have you ever seen or signed up to any websites, social media, forums, baby clubs etc that are linked to formula brands?

40. Have you ever received any free samples of formula? If so where from?
    - *Was this in hospital? Or outside of hospital?*
    - *Which brand?*
    - *Why were you given a sample?*

41. Have you ever been contacted directly by a formula company?

## SECTION EIGHT: SUMMING UP

42. Do you think there is enough support available for mothers with regards to feeding their babies? What else is needed?

43. What do you think women want to hear about infant feeding? About breastfeeding, formula feeding?

44. What don't they want to hear? About breastfeeding, formula feeding?

45. If there was advertising to promote breastfeeding – what should it say, who should it feature – celebrities, ordinary mums etc

MODERATOR - READ THE FOLLOWING

*This brings us to the end of the discussion. Thank you very much for taking the time to be here today.*

*Does anyone have anything further to add, or is there anything anyone wants to ask me?*

*As stated previously your data will be stored securely. Your anonymised data will be shared with the World Health Organization for public health research purposes. Are you happy with this?*

**[INTERVIEWER - COLLECT VERBAL CONSENT (Y/N).**

**Further questions**

If you have any further questions please do not hesitate to ask the researcher before, during, or after the interview is completed.

Please direct any research enquiries to the KLA research team:

Robert Baczynski or Khomotso Kumalo
Landline: 011 447 8411
Email: robertb@kla.co.za or Khomotsok@kla.co.za

If you have any questions or concerns about how to feed or care for your baby, please contact your health professional or the following regional services:

**Support Services Information**

**Gender Abuse**

1. POWA (People opposed to Woman abuse): 011 6424345
2. Stop Gender Abuse: 0800150150

**Child Abuse**

1. Childline 0740808315

**Depression / psychological support**

1. Life line 011 7281347
2. WhatsApp call counselling line 0659899238

**Breastfeeding support**

1. La Leche League of South Africa

Western Cape: 021 855 4657
Gauteng: 012 332 2564

2. Breastfeeding Association

Gauteng: 011 883 9873
Western Cape: 021 887 4213
